# Effective identification of cancer predisposition syndromes in children with cancer employing a questionnaire

**DOI:** 10.1007/s10689-021-00233-5

**Published:** 2021-03-02

**Authors:** Miriam Schwermer, Astrid Behnert, Beate Dörgeloh, Tim Ripperger, Christian P. Kratz

**Affiliations:** 1 Pediatric Hematology and Oncology, Hannover Medical School, Hannover, Germany; 2 Department of Human Genetics, Hannover Medical School, Hannover, Germany; 3 Rare Disease Program, Hannover Medical School, Hannover, Germany

**Keywords:** Cancer predisposition syndromes, Questionnaire, Pediatric cancer

## Abstract

Approximately 10% of children with newly diagnosed cancer have a cancer predisposition syndrome (CPS). The optimal diagnostic approach to identify them among children diagnosed with cancer is unknown. *Objective*: To determine whether the use of a one-page questionnaire can improve the CPS diagnosis among children with an oncologic condition. *Design*: Comparative effectiveness research. *Setting*: Referral center for children with cancer. *Results*: 739 children diagnosed with an oncologic condition between 2012 and 2019. All children with a newly diagnosed oncologic condition presenting to Hannover Medical School between January 1st 2017 and December 31st 2019 were prospectively evaluated with a CPS questionnaire. Children in whom the questionnaire suggested the need of a genetic workup were further evaluated. All children diagnosed with an oncologic condition between January 1st 2012 and December 31st 2016 served as control. The CPS diagnoses established during both time periods were evaluated and compared. A CPS was diagnosed in 27 out of 287 children (9.4%) during the *questionnaire* period versus 24 out of 452 children (5.3%) during the *control* period (*P* = 0.032). *Conclusion*: The CPS questionnaire appears to significantly improve the diagnosis of children with CPS among children with a newly diagnosed oncologic condition.

**Supplementary Information:**

The online version contains supplementary material available at 10.1007/s10689-021-00233-5.

## Introduction

Cancer predisposition syndromes (CPS) are a major cause of childhood cancer. Several next generation sequencing (NGS) studies have shown that the proportion of children with cancer who have a CPS is larger than previously anticipated [1–3]. Given the clinical relevance of a CPS in a child with cancer (e.g., counseling, psychologic support, prevention, surveillance, treatment, and identification of relatives at risk), a small number of centers screen for the presence of a CPS by offering a genetic evaluation and (epi)genetic testing of germline DNA to all patients; however, this resource is only available to a small number of centers or to children with selected entities (e.g., in Germany, all children with brain tumors are currently being offered testing through the brain tumor studies).

In order to guide pediatric oncologists to decide which patients have a high probability of an underlying CPS and would benefit from genetic counseling and testing, we and others have developed questionnaires and mobile apps [4–7]. Based on clinical features, previous cancer (family) history, cancer sub-type, and somatic mutational spectrum, it is decided on whether a genetic evaluation is indicated. Here, we show that use of one of such tools [5] is associated with a significant increase of CPS diagnoses among children with a newly diagnosed oncologic condition.

## Methods

The previously described questionnaire (see Supplement and reference [5]) originally developed by Jongmans and colleagues [4] and updated by the cancer predisposition working group of the German Society of Pediatric Oncology and Hematology with input from various trial groups [5] was prospectively employed in all 287 children presenting with an oncologic condition to Hannover Medical School during a 3-year period (i.e., 2017–2019). All children who were diagnosed with an oncologic condition within the prior 5-year period when the questionnaire was not applied (i.e., 2012–2016, n = 452) served as control. Children with a questionnaire result indicating the presence of a CPS (i.e., ≥ 1 fulfilled criterion from the questionnaire) were further evaluated by a CPS specialist (i.e., an oncologist with expertise in genetics or a geneticist with expertise in cancer predisposition) to determine whether further genetic testing was warranted. Only if this initial genetic evaluation revealed that the genetic testing criteria of a known CPS were met, genetic counselling and testing was offered. The CPS diagnoses established during the questionnaire and control periods were compared. We employed Pearson’s χ^2^ test and a *P* value lower than 0.05 was regarded as statistically significant. The study was approved by the ethical review board at Hannover Medical School.

## Results

**Fig. 1:**
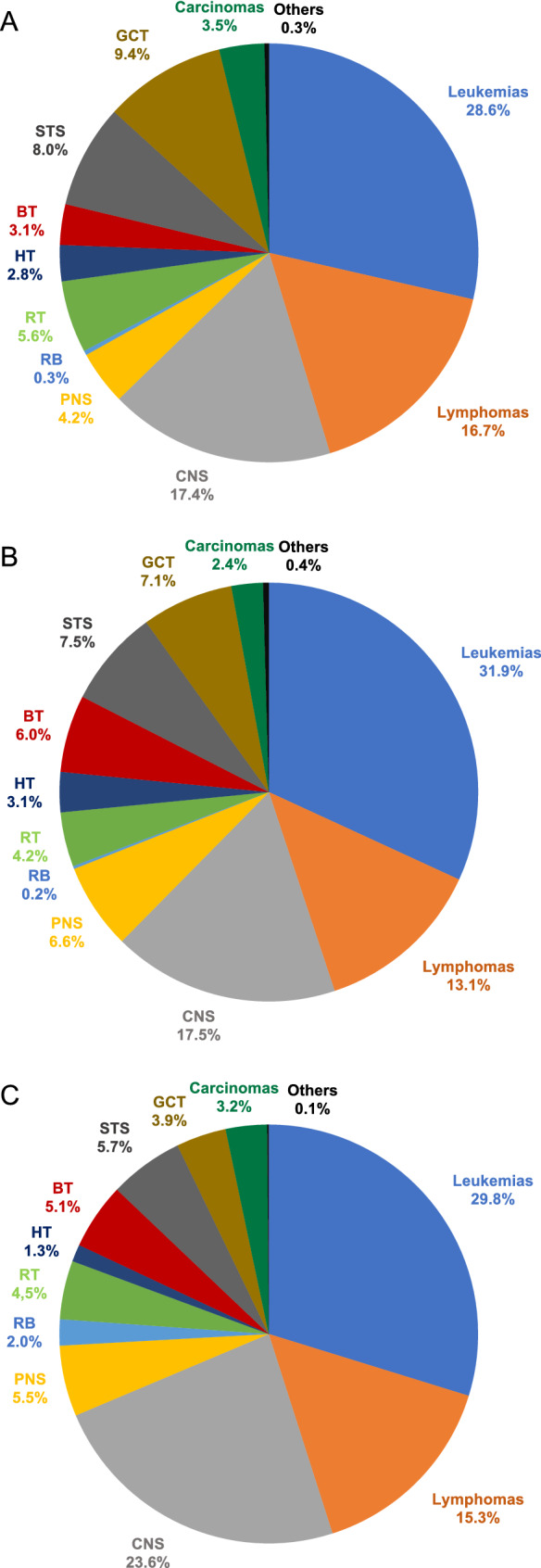
Relative frequencies of pediatric cancer types. **a** Shows the distribution of pediatric cancer types diagnosed at Hannover Medical School between 2017 and 2019 (n = 287); **b** shows the distribution of pediatric cancer types diagnosed at Hannover Medical School between 2012 and 2016 (n = 452); **c** shows the distribution of pediatric cancer types reported to the German Childhood Cancer Registry between 2009 and 2018 (n = 21,831) [8]. *BT* bone tumors, *GCT* germ cell tumors, *HT* hepatic tumors, *PNS* peripheral nervous cell tumors, *RB* retinoblastoma, *RT* renal tumors, *STS* soft tissue sarcomas

Figure 1 depicts the distribution of pediatric cancer types diagnosed at Hannover Medical School within the *questionnaire* and the *control* periods. The cancer distribution during both study periods are similar and resemble the pediatric cancer spectrum captured by the German Childhood Cancer Registry between 2009 and 2018 [8]. In 86 out of 287 children (30%) the questionnaire indicated a high likelihood of an underlying CPS. After expert review, 20 of the 86 patients were not further evaluated because the clinical constellation appeared unlikely to be associated with a currently known CPS (e.g., the questionnaire was positive but the testing criteria for a known CPS were not met). Of the remaining 66 patients, 3 declined further evaluation, 3 were not evaluated due to the patient’s death or the family’s relocation. The remaining 60 patients were offered counseling and testing and a CPS was diagnosed (or known prior to the cancer diagnosis) in 27 patients based on germline testing (9.4% of the entire group and 45% of the patients that were offered counseling and testing). In contrast, among the 452 patients who were diagnosed with an oncologic condition during the control period, a CPS diagnosis was established (or known prior to the cancer diagnosis) in 24 patients (5.3%). When comparing both groups, the number of patients diagnosed with a CPS was significantly higher during the questionnaire period than the number of CPS patients diagnosed during the control period (*P* = 0.032). It can be assumed that all patients in whom the CPS diagnosis was established prior to the cancer diagnosis or presentation to our department (e.g., Down syndrome, Neurofibromatosis type 1 would have been detected clinically when the patients presented with the oncologic condition. Nevertheless, conservatively excluding these CPS patients from the analysis, the difference remains significant. After exclusion of these previously known CPS cases, 13 CPS among 287 patients were diagnosed during the questionnaire and 7 CPS among 452 patients during the control period (*P* = 0.015). Tables 1 and 2 show details on individual CPS patients diagnosed during both periods. Notably, one patient suffered from a mitochondrial liver disease caused by germline defects of *TRMU* [9]. Although this condition is not an established CPS, we assume that the liver tumor that occurred in that patient was caused by the underlying liver condition. Four patients have been described elsewhere [10–13].

**Table 1:** Individuals diagnosed with CPS employing the screening tool (2017–2019)

No.	Cancer	A@D	Sex	Reason for evaluation	Genetic cause	CPS
1^b^	ALL	3.4	M	Physical features	Trisomy 21	DS
2^b^	ALL	6.1	M	Physical features developmental delay	*ATM*, c.3576G > A p. (Ser1135_Lys1192del58), homozygous, aberrant splicing	AT
3	CN	1.1	M	Pathology	arr [GRCh37] 14q32.12q32.2 (9450372296382117) × 1, deletion including *DICER1*	DICER1 syndrome
4^b^	FH	0.3	M	Physical features	*NF1*, c.4812C > A p. (Tyr1604*)	NF1
5	GIST	14.11	M	Pathology	*SDHA*, c.688del p. (Glu230Serfs*10)	HPPS
6	GB	9.9	F	Physical features, consanguinity, pathology	*MSH6*, c.691delG p. (Val231Tyrfs*15), homozygous	CMMRD
7^b^	GB	11.0	F	Physical features, consanguinity, pathology	*MSH6*, c.691delG p. (Val231Tyrfs*15), homozygous	CMMRD
8	GB	12.11	M	Physical features, pathology	*MSH6,* c.691del p. (Val231Tyrfs*15) and c.2906A > G p. (Tyr969Cys), compound heterozygous	CMMRD
9^b^	Glioma	15.6	F	Physical features	*NF1*, c.6819 + 3A > T p.?, VUS	NF1
10^b^	OPG	3.8	F	Physical features	*NF1,* c.3822_3823del p. (Phe1275Profs*8)	NF1
11^b^	OPG	9.2	M	Physical features	Work-up pending	NF1^a^
12^b^	OPG, MPNST	10.1	F	Physical features	Work-up pending	NF1^a^
13^b^	HB	1.0	F	Physical features	*KCNQ1OT1*: TSS DMR LOM (IC2 LOM)	BWS [10]
14^b^	HB	11.6	F	Metabolic features	*TRMU,* c.653G > T p. (Ser218Ala) and c.1081_1082insAGGCTGTGC, p. (Arg361Ala Val Arg), compound heterozygous	Liver failure, transient infantile
15	MG	8.0	F	Pathology	*SMARCE1*, c.959delC p. (Pro320Leufs*122)	SMARCE1-related meningioma
16	MG	15.11	M	Pathology	*BAP1*, c.2056 + 1G > A r.2056_2057ins180 p.Gly687Glufs*30	BAP1 tumor predisposition syndrome
17	MDS	3.1	F	Immunodeficiency, physical features, hematology, cytogenetics	*SAMD9*, c.4690G > C p. (Gly1564Arg), VUS	MIRAGE syndrome^a^
18^b^	MDS	15.9	M	Hematology	*HAX1,* c.130_131insA p. (Trp44*)	SCN [11]
19	MDS	17.4	F	Family history, pathology	*GATA2*, c.1186C > T p. (Arg396Trp)	GATA2 deficiency
20	WT	0.7	F	Lateralized overgrowth, pathology	upd(11)pat	BWS
21	MDS	1.9	F	Immunodeficiency, physical features, hematology, cytogenetics	*SAMD9L*, c.3584C > T p. (Ala1195Val)	Ataxia-pancytopenia syndrome
22	RT	1.1	F	Pathology	nuc ish 6 (CEP6 × 2), 22 (RP11-71G19 × 1, RP11-911F12 × 1), heterozygous *SMARCB1* deletion	RTPS
23	SEGA	6.4	F	Physical features, pathology	*TSC2*, c.1513C > T p. (Arg505*)	TSC
24^b^	TMPD	0.2	F	Physical features, hematology	*PTPN11*, c.182A > G, p. (Asp61Gly)	NS
25^b^	TMPD	0.0	F	Physical features	Trisomy 21	DS
26^b^	TMPD	0.0	F	Physical features	Trisomy 21	DS
27	Teratoma	0.11	M	Physical features	*MNX1*, c.53delC p. (Pro18Hisfs*204)	Currarino syndrome

*A@D* age in years at cancer diagnosis, *ALL* acute lymphoblastic leukemia, *AT* ataxia teleangiectasia, *BWS* Beckwith Wiedemann syndrome, *CALS* café-au-lait spots, *CMMRD* constitutional mismatch repair deficiency, *CN* cystic nephroma, *DS* Down syndrome, *FH* fibrous histiocytoma, *GB* glioblastoma, *GIST* gastrointestinal stromal tumor, *HB* hepatoblastoma, *HPPS* hereditary pheochromocytoma/paraganglioma syndrome, *IC2 LOM* imprinting center 2 loss of methylation, *MDS* myelodysplastic syndrome, *MG* meningioma, *MPNST* malignant peripheral nerve sheet tumor, *NF1* Neurofibromatosis type 1, *NS* Noonan syndrome, *OPG* optic pathway glioma, *RT* rhabdoid tumor, *RTPS* rhabdoid tumor predisposition syndrome, *SCN* severe congenital neutropenia, *SEGA* subependymal giant cell astrocytoma, *TMPD* transient myeloproliferative disease, *TSC* tuberous sclerosis, *TSS DMR LOM* transcription start site differentially methylated region, *upd(11)pat* paternal uniparental isodisomy of 11p15.5, *VUS* variant of uncertain significance (ACMG class 3), *WT* nephroblastoma

^a^Clinically confirmed CPS diagnosis

^b^CPS diagnosis was known prior to the oncologic diagnosis or presentation to Hannover Medical School

**Table 2:** Individuals diagnosed with CPS before the screening tool was introduced (2012–2016)

No.	Cancer	Sex	A@D	Reason for evaluation	Genetic cause	CPS
1^b^	ALL	F	7.7	Physical features	Trisomy 21	DS
2^b^	AML	M	1.0	Physical features	Trisomy 21	DS
3^b^	AML	M	3.1	Physical features	Trisomy 21	DS
4^b^	AML	F	3.1	Physical features	Trisomy 21	DS
5^b^	AML	M	3.8	Physical features	Trisomy 21	DS
6	AML	F	11.7	Physical features	*FANCA*, c.45G > A p. (Trp15*), and c.67delG p. (Asp23Ilefs*23), compound heterozygous	FA
7	CRC	M	14.3	Physical features, pathology	*POLE*, c.1231G > C p. (Val411Leu)	POLE deficiency [12]
8^b^	OPG	F	4.6	Physical features	Work up pending	NF1^a^
9^b^	OPG	F	6.9	Physical features	Work up pending	NF1^a^
10^b^	OPG	M	12.9	Physical features	Work up pending	NF1^a^
11	OPG	F	1.4	Physical features	Work up pending	NF1^a^
12^b^	OPG	F	6.7	Physical features	Work up pending	NF1^a^
13^b^	MPNST	F	6.6	Physical features	Work up pending	NF1^a^
14^b^	HD	M	11.7	Immunodeficiency	*PIK3CD*, c.1689 + 9G > A and c.3061G > A p. (Glu1021Lys), compound heterozygous	Activated PIK3CD syndrome
15	MDS	F	13.3	Pathology	*FANCA*, c.1814_1815delAG p. (Glu605Valfs*7)	FA
16	NBL	F	0.11	Pathology	*ALK*, c.3824G > A p. (Arg1275Gln)	NBL predisposition
17^b^	RMS	F	2.2	Family history	*TP53*, c.309C > G p. (Tyr103*)	LFS
18	TT	F	12.2	Pathology	*DICER1*, c.2920dupA p. (Thr974Asnfs*6)	DICER1 syndrome
19^b^	TMPD	M	0.0	Physical features	Trisomy 21	DS
20^b^	TMPD	M	0.0	Physical features	Trisomy 21	DS
21^b^	TMPD	M	0.2	Physical features	Trisomy 21	DS
22^b^	TMPD	M	0.2	Physical features	Trisomy 21	DS
23^b^	RB	F	1.10	Physical features	arr [GRCh37] 13q14.13q21.33 (45943304_68903406) × 1	13q deletion syndrome
24	cMX	M	15.1	Pathology	arr [GRCh37] 17q24.2 (66501525_66512418) × 1	Carney Complex [13]

*A@D* age in years at cancer diagnosis, *ALL* acute lymphoblastic leukemia, *CALS* café-au-lait spots, *CRC* colorectal carcinoma, *DS* Down syndrome, *FA* Fanconi anemia, *HD* Hodgkin disease, *LFS* Li Fraumeni syndrome, *MDS* myelodysplastic syndrome, *MPNST* malignant peripheral nerve sheet tumor, *cMX* cardial myxoma, *NBL* neuroblastoma, *NF1* neurofibromatois type 1, *OPG* optic pathway glioma, *RB* retinoblastoma, *RMS* rhabdomyosarcoma, *TT* thyroid tumor, *TMPD* transient myeloproliferative disease

^a^Clinically confirmed CPS diagnosis

^b^CPS diagnosis was known prior to the oncologic diagnosis or presentation to Hannover Medical School

## Discussion

Here, we show that the systematic use of a CPS questionnaire [5] was associated with a significant increase of CPS diagnoses among children with a newly diagnosed oncologic condition. The proportion of children diagnosed with a CPS using this clinical approach resembles the proportion of children diagnosed by (epi)genetic testing [1–3], suggesting that not many children with a CPS are being overlooked using this approach. However, in order to define the negative and positive predictive values and sensitivity/specificity of the questionnaire the study design would need to include both, agnostic (epi)genetic testing and the questionnaire. The questionnaire approach, by definition, misses children with hidden or atypical CPS features (e.g., a patient with Li-Fraumeni syndrome with a de novo variant in *TP53* and osteosarcoma would not be detected through this approach). Also, children with subtle features of a CPS may be missed if patients are not evaluated by an experienced dysmorphologist. Most patients in whom a CPS diagnosis was established had oncologic conditions that by itself suggested the presence of a CPS diagnosis when observed in childhood (e.g., cystic nephroma, meningioma, gastrointestinal stromal tumor, myelodysplastic syndrome) or obvious physical features leading to the CPS diagnosis (e.g., lateralized overgrowth).

One potential advantage of the questionnaire approach is the preferential identification of children with a clinically relevant CPS. In contrast, a genetic evaluation and agnostic (epi)genetic testing offered to all children with cancer has the probability of identifying gene variants in known or scientifically suspected CPS genes with unknown clinical relevance (e.g., heterozygous variants in recessive cancer genes or variants in cancer genes predisposing to malignancy during adulthood). While this knowledge is of high scientific interest, it may not influence the clinical care and may have potential adverse effects (anxiety, costs).

The study has several limitations: (1) The study took place in a center with special interest in CPS. Thus, the CPS diagnoses during both time periods may have been influenced and improved by this expertise. This factor may have led to the observation that even in the control period, rare CPS were identified [12, 13]. (2) A further genetic evaluation was initiated only in situations when it appeared likely that a known CPS could explain the clinical situation. Thus, the likelihood of making novel discoveries was decreased. (3) Several patients were diagnosed with a CPS prior to the development of cancer, however, when we exclude these patients from the analysis, the results remained significant. (4) We cannot rule out that the study is influenced by coincidental factors, for example, a small number of additional cancer types highly associated with a CPS during the control period may have led to different results. (5) The list of CPS as well as awareness are constantly growing [14–16]. These factors could have led to more CPS diagnoses during the later questionnaire period.

## Conclusion

Despite these limitations, our data suggest that tools like a CPS questionnaire may significantly improve the diagnosis of CPS among children with cancer. Although negative and positive predictive values and sensitivity/specificity are unknown, it is likely that a small number of cases of CPS will be missed using clinical approaches.

## Supplementary Information

Below is the link to the electronic supplementary material.

Supplementary file 1 (DOCX 28 KB)

## Data Availability

Raw data and material and processed data are held within the Department of Pediatric Hematology and Oncology at Hannover Medical School.

## References

[1] Sylvester DE, Chen Y, Jamieson RV, Dalla-Pozza L, Byrne JA (2018). Investigation of clinically relevant germline variants detected by next-generation sequencing in patients with childhood cancer: a review of the literature. J Med Genet.

[2] Zhang J, Walsh MF, Wu G (2015). Germline mutations in predisposition genes in pediatric cancer. N Engl J Med.

[3] Grobner SN, Worst BC, Weischenfeldt J (2018). The landscape of genomic alterations across childhood cancers. Nature.

[4] Jongmans MC, Loeffen JL, Waanders E (2016). Recognition of genetic predisposition in pediatric cancer patients: an easy-to-use selection tool. Eur J Med Genet.

[5] Ripperger T, Bielack SS, Borkhardt A (2017). Childhood cancer predisposition syndromes-A concise review and recommendations by the Cancer Predisposition Working Group of the Society for Pediatric Oncology and Hematology. Am J Med Genet A.

[6] Goudie C, Cullinan N, Villani A (2018). Retrospective evaluation of a decision-support algorithm (MIPOGG) for genetic referrals for children with neuroblastic tumors. Pediatr Blood Cancer.

[7] Hopman SM, Merks JH, de Borgie CA (2013). The development of a clinical screening instrument for tumour predisposition syndromes in childhood cancer patients. Eur J Cancer.

[8] Erdmann F, Kaatsch P, Grabow D, Spix C (2020) German childhood cancer registry: annual report 2019 (1980–2018). Institute of Medical Biostatistics, Epidemiology and Informatics (IMBEI) at the University Medical Center of the Johannes Gutenberg University Mainz 2020

[9] Zeharia A, Shaag A, Pappo O (2009). Acute infantile liver failure due to mutations in the TRMU gene. Am J Hum Genet.

[10] Coktu S, Spix C, Kaiser M (2020). Cancer incidence and spectrum among children with genetically confirmed Beckwith-Wiedemann spectrum in Germany: a retrospective cohort study. Br J Cancer.

[11] Klein C, Grudzien M, Appaswamy G (2007). HAX1 deficiency causes autosomal recessive severe congenital neutropenia (Kostmann disease). Nat Genet.

[12] Wimmer K, Beilken A, Nustede R (2017). A novel germline POLE mutation causes an early onset cancer prone syndrome mimicking constitutional mismatch repair deficiency. Fam Cancer.

[13] Behnert A, Ripperger T, Jack T, Franke D, Horke A, Kratz C (2016). Linksatriale Raumforderung und Lentigines. Monatsschr Kinderheilkd.

[14] Chen DH, Below JE, Shimamura A (2016). Ataxia-pancytopenia syndrome is caused by missense mutations in SAMD9L. Am J Hum Genet.

[15] Narumi S, Amano N, Ishii T (2016). SAMD9 mutations cause a novel multisystem disorder, MIRAGE syndrome, and are associated with loss of chromosome 7. Nat Genet.

[16] Waszak SM, Robinson GW, Gudenas BL (2020). Germline Elongator mutations in Sonic Hedgehog medulloblastoma. Nature.

