# Supplementary material for: Effective identification of cancer predisposition syndromes in children with cancer employing a questionnaire
Source: Fam Cancer. 2021 Mar 2;20(4):257–62. doi: 10.1007/s10689-021-00233-5 (PMC8484089; doi:10.1007/s10689-021-00233-5)
Supplement: Supplementary file 1 — (DOCX 28 KB) [file 10689_2021_233_MOESM1_ESM.docx]

**Childhood cancer: Indication for genetic counseling?***

*updated Jongmans criteria [[Jongmans et al., 2016](#_ENREF_1)]

*if at least one criterion is fulfilled, your patient may benefit from genetic counseling*

**1. Family history (3 generation pedigree)**

O ≥2 malignancies occurred in family members before age 18 years, including index patient

O Parent or sibling with current or history of cancer before age 45 years

O ≥2 first or second degree relatives in the same parental lineage with cancer before age 45 years

O The parents of the child with cancer are consanguineous

**2. One of the following Neoplasms was diagnosed:**

O Adrenocortical carcinoma / adenoma

O ALL (low hypodiploid)

O ALL (ring chromosome 21)

O ALL (Robertsonian translocation 15;21)

O ALL relapse (*TP53* mutated)

O AML (Monosomy 7)

O Basal cell carcinoma

O Botryoid rhabdomyosarcoma of the urogenital tract (fusion-negative)

O Chondromesenchymal harmatoma

O Choroid plexus carcinoma / tumor

O Colorectal carcinoma

O Cystic nephroma

O Endolymphatic sack tumor

O Fetal rhabdomyoma

O Gastrointestinal stromal tumor

O Glioma of the optic pathway (with signs of NF1)

O Gonadoblastoma

O Hemangioblastoma

O Hepatoblastoma (*CTNNB1* wildtype)

O Hepatocellular carcinoma

O Infantile myofibromatosis

O Juvenile myelomonocytic leukemia

O Keratocystic odontogenic tumor

O Large cell calcifying Sertoli-cell-tumor

O Malignant peripheral nerve sheath tumor

O Medullary thyroid carcinoma

O Medulloblastoma (SHH activated)

O Medulloblastoma (WNT activated, *CTNNB1* wildtype)

O Medullary renal cell carcinoma

O Medulloepithelioma

O Melanoma

O Meningioma

O Myelodysplastic syndrome

O Myeloproliferative neoplasms (except CML)

O Myxoma

O Neuroendocrine tumor

O Paraganglioma / pheochromocytoma

O Parathyroid carcinoma / adenoma

O Pineoblastoma

O Pituitary adenoma / tumor

O Pituitary blastoma

O Pleuropulmonary blastoma

O Renal cell carcinoma

O Retinoblastoma

O Rhabdoid tumor

O Rhabdomyosarcoma with diffuse anaplasia

O Schwannoma

O Schwannomatosis

O Sertoli-Leydig cell tumor

O Sex cord stromal tumor with annular tubules

O Small cell carcin. of the ovary hypercalcemic type

O Squamous cell carcinoma

O Subependymal giant cell astrocytoma

O Thyroid carcinoma (non-medullary)

O Transient myeloproliferative disease

**O Other rare cancers or cancers that typically occur in adults, unusually early manifestation age**

**3.** O **Genetic tumor analysis reveals defect suggesting a germline predisposition**

**4.** O **A patient with ≥2 malignancies (**e.g. secondary, bilateral, multifocal, metachronous**)**

**5.** O **A child with cancer and congenital or other anomalies**

| ***Sign*** | ***Think of*** |
| --- | --- |
| O Congenital anomalies | Abnormal organs, skeletal anomalies, oral clefting, abnormal teeth, urogenital anomalies, abnormal hearing or vision, etc. |
| O Facial dysmorphism |  |
| O Mental impairment, developmental delay | Abnormal behavior, learning difficulties |
| O Abnormal growth | Height, head circumference, birth weight, hemihyperplasia, growth chart |
| O Skin anomalies | Abnormal pigmentation such as ≥2 café-au-lait spots, vascular lesions, hypersensitivity to sun, benign tumors, etc. |
| O Hematological abnormalities (not explained by current cancer) | Pancytopenia, anemia, thrombocytopenia, neutropenia, leukopenia, macrocytic erythrocytes |
| O Immune deficiency | Frequency of infections, lymphopenia |
| O Endocrine anomalies | Primary hyperparathyroidism, precocious puberty, gigantism/acromegaly, Cushing syndrome |

**6.** O **The patient suffers from excessive toxicity of cancer therapy**
